# Impact of a Time-Related Benchmark on the Adenoma Detection Rate in Surveillance Colonoscopy: A STROBE Statement-Oriented Cross-Sectional Cohort Study

**DOI:** 10.5152/tjg.2023.22883

**Published:** 2023-12-01

**Authors:** Tesshin Ban, Yoshimasa Kubota, Tomonori Yano, Makiko Naka Mieno, Takuya Takahama, Shun Sasoh, Satoshi Tanida, Tomoaki Ando, Makoto Nakamura, Takashi Joh

**Affiliations:** 1 Department of Gastroenterology and Hepatology, Gamagori City Hospital, Gamagori, Japan; 2 Division of Medicine, Deparment of Gastroenterology, Jichi Medical University, Shimotsuke, Japan; 3 Department of Medical Informatics, Center for Information, Jichi Medical University, Shimotsuke, Japan

**Keywords:** Adenoma, benchmarking, colonoscopy

## Abstract

**Background/Aims:**

Colorectal adenomas are precursor lesions of globally increasing colorectal cancer. Hence, a high adenoma detection rate in colonoscopy is pivotal. We investigated the clinical impact of stratified colonoscopy observation time combined with observation time/intubation time ratio on the detection of colorectal adenomas.

**Materials and Methods:**

We conducted a single-center retrospective study including 369 consecutive patients who underwent colonoscopy following fecal immunochemical tests between May 2021 and April 2022. The primary outcome measure was the impact of the stratified observation time and observation time/ intubation time ratio (category 1: <6.0 minutes and <1.0, category 2: <6.0 minutes and ≥1.0, category 3: ≥6.0 minutes and <1.0, and category 4: ≥6.0 minutes and ≥1.0) on adenoma detection rate.

**Results:**

Cecum intubation was obtained in 367 patients (99.5%). Adenomas were detected in 226 patients (61.2%). From the univariate analysis, age ≥53 years, habitual alcohol intake, colonoscopy attachment (+), and observation time with observation time/intubation time ratio categories 3 and 4 were determined as significant factors for adenoma detection rate. From the logistic regression analysis, age ≥ 53 years (odds ratio: 4.86, 95% CI: 2.25-10.52), habitual alcohol intake (odds ratio: 2.26, 95% CI: 1.33-3.82), category 3 (odds ratio: 3.66, 95% CI: 1.81-7.45), and category 4 (odds ratio: 5.60, 95% CI: 2.92-10.73) were significant factors for adenoma detection rate.

**Conclusion:**

We propose the observation time with observation time/intubation time ratio combined benchmark (with categories’ thresholds based on observation time >6 minutes and scope withdrawal time exceeding intubation time [observation time/intubation time ratio > 1]) as a novel colonoscopy quality indicator. These findings represent an important educational message for endoscopists.

**Main Points**

- An adenoma is a precursor lesion of colorectal cancer. Therefore, improving the adenoma detection rate at colonoscopy (CS) is pivotal.
- Colonoscopy observation time ≥6 minutes is the only well-known time-related benchmark. However, CS observation time depends on various factors.
- Colonoscopy observation time ≥6 minutes has an additional positive clinical impact on the colorectal adenoma detection rate when it exceeds the intubation time.

## Introduction

Colorectal cancer (CRC) is one of the most lethal neoplasms. Globally, CRC is the third most frequent malignancy and the second most common cause of cancer death.^1, 2^ The prevalence of CRC in the world was 1.9 million in 2020 and is projected to increase to 3.1 million in 2040. This rising trend is predicted all over the world^2^ and, to counteract this, surveillance for CRC is pivotal. An annual fecal immunochemical test (FIT) followed by a colonoscopy (CS) is recommended as 2-step surveillance for the average-CRC risk population over 45 years.^1, 3, 4^ The sensitivity of FIT for CRC is as high as 91.0% but low for adenoma and sessile serrated lesions (SSLs; 6.0%-56.0% and 5.0%-16.0%, respectively).^4^ In addition to the adenoma-carcinoma sequence, 10%-15% of CRC derive from the SSL-to-carcinoma transition.^1^

A high adenoma detection rate (ADR) in CS is crucial in reducing the mortality rate caused by CRC.^5^ However, 26.0% of diminutive (under 5 mm) polypoid adenomas are missed during CS; therefore, providing an accurate CS examination is warranted.^6^ In a Polish study, colonoscopies with ADRs under 20% had an over 10-fold higher rate of post-CS CRC than those with higher ADRs.^7^ This may underlie the recommended minimum threshold of 25% ADR overall for screening colonoscopy.^8^ In another study, a 1% increase in ADR was associated with a 3% reduction in CRC incidence and a 5% reduction in CRC-related death.^5^ It has been shown that a CS withdrawal phase ≥6 minutes was the threshold between relatively high and low ADRs.^9^ Therefore, to date, quality indicators for CS include ADR ≥25%, cecum intubation rate ≥95%, and observation time (t-OBS) ≥6 minutes.^4^ However, time-related indicators, including intubation time (t-INT) and t-OBS are influenced by endoscopist-related factors (e.g., daily time frame, skill level, or personality), and by the patient-related factors (e.g., colon length or previous surgery). Therefore, when used alone, these time-related indicators entirely depend on these factors in daily practice. Thus, we focused on t-OBS/t-INT ratio in addition to t-OBS, hypothesizing that there would be a significant clinical impact of the stratified t-OBS with t-OBS/t-INT combined benchmark on the detection of colonic adenomas.

## Materials and Methods

### Study Design

This study is a single-center, cross-sectional cohort study based on the strengthening the reporting of observational studies in epidemiology (STROBE) guidelines.

### Study Population

After the institutional review board of Gamagori City Hospital approved the protocol, we reviewed our CS database between May 2021 and April 2022.

Patient inclusion criteria were as follows: (i) age ≥20 years, (ii) undergoing FIT and (iii) CS, and (iv) providing written consent for CS.

Patient exclusion criteria were as follows: (i) previous colorectal resection, (ii) an already known colonic malignancy, (iii) insufficient bowel preparation (e.g., by enema only), and (iv) unavailable data for the primary endpoint.

### Examination Methods

Patient/CS characteristics and detected tumorous lesions with a final diagnosis were prospectively and consecutively recorded in our institutional CS database. Patient characteristics included age, sex, time from previous CS, FIT result, previous abdominal operation, smoking/drinking status, antiplatelet/anticoagulant use, and body mass index (BMI). The CS characteristics included endoscopist skills, the use of antispasmodic agents and/or sedative agents, bowel preparation, Boston bowel preparation scale (BBPS) score, CS attachment, cecum intubation, t-INT, t-OBS, intervention time, total time, and adverse events. If the intubation by the trainee endoscopist stagnated for 10 minutes, the senior endoscopist took over as needed. Meanwhile, observations by the trainee were performed under the supervision of the senior endoscopist. A trainee was defined as an endoscopist who began learning CS during the study period. Antispasmodic agents, prioritizing scopolamine butylbromide (20 mg, intramuscular injection), were administrated during most procedures. Moderate sedation under midazolam (0.03-0.04 mg/kg) plus pentazocine (7.5-15.0 mg/kg)^10, 11^ was allowed and performed according to the patients’ wishes. Bowel preparation was performed using a sodium–potassium-based colon cleansing product. The BBPS score was recorded according to previous studies.^12, 13^ The use of a simple CS attachment^14^ was left at the operator’s discretion. Adverse events were recorded according to the lexicon for endoscopic adverse events.^15^ In this study, investigations were performed using various colonoscopes under CO_2 _insufflation; however, neither water immersion/exchange techniques nor artificial intelligence detection software was employed.^16, 17^

Detected tumorous lesions were recorded with macroscopic findings. All these lesions were pathologically confirmed using the specimen obtained by endoscopic intervention or operation. Macroscopic findings of colonic neoplasm were classified based on the Paris classification.^18^ The endoscopic intervention included biopsy, cold forceps polypectomy (CFP), cold snare polypectomy (CSP), endoscopic mucosal resection (EMR), and endoscopic submucosal dissection (ESD).^19, 20^ We captured precise endoscopic milestone timestamps at anal scope intubation, cecum intubation, beginning and end of each intervention, and anal extubation. Accordingly, time-related indicators could be calculated by subtracting between these milestone times. In addition, we allowed some endoscopic interventions, including biopsy, CFP, and CSP, during the scope extubation phase, but not during the intubation phase. The EMR and ESD were rescheduled for the following day.

### Outcome Measures

The primary outcome measure was the impact of the stratification by combined time-related indicators, including t-OBS and t-OBS/t-INT ratio, on ADR. The secondary outcome measures were the impact of the stratified time-related indicators on the number of adenomas detected and the statistical power analysis regarding the primary outcome measure.

### Statistical Analysis

Categorical variables were dichotomized, as follows: smoking status (never smoked or ex-smoker/current smoker), drinking (not-habitual/habitual), and endoscopist skill (not-trainee/trainee). Continuous variables were described with mean ± SD. Then, an appropriate cutoff value in the continuous variables was calculated using the receiver operating characteristic (ROC) curve with the Youden index. However, an already-known cutoff value of t-OBS ≥6.0 minutes^4, 9^ was used in this analysis. Potential factors affecting ADR, including age, sex, FIT result, previous CS, previous abdominal surgery, smoking/drinking status, BMI, antiplatelet/coagulant, endoscopist skill, sedation, antispasmodic agents, CS attachment, and time-related indicators stratified by t-OBS with t-OBS/t-INT ratio were analyzed using the chi-square test or Fisher’s exact test. Finally, significant factors for ADR were analyzed by logistic regression analysis after controlling for potential confounders and expressed as odds ratio (OR) with a 95% CI. The number of adenomas by each time-related indicator category was analyzed using the Kruskal–Wallis test followed by the Bonferroni correction. No a priori statistical sample size calculation was conducted. Therefore, a post-hoc-statistical power analysis regarding the primary outcome measure was conducted as part of the secondary outcome measures. IBM Statistical Package for the Social Sciences Statistics 28 (IBM Japan, Ltd., Tokyo, Japan) and G*power 3.1 software (https://www.psychologie.hhu.de/arbeitsgruppen/allgemeine-psychologie-und-arbeitspsychologie/gpower) were used for statistical analyses.

### Ethical Statement

This study was conducted in accordance with the ethical standards of the responsible committee on human experimentation and the Helsinki Declaration of 1964 and later versions. We obtained written consent from all patients for the CS investigation, and an opt-out approach was used to obtain consent for the publication of this study. This study was approved by the ethics committee of Gamagori City Hospital (approval number: 209-1) and registered in the UMIN individual case data repository (UMIN000049187).

## Results

### Characteristics of the Participants

**Figure 1.**
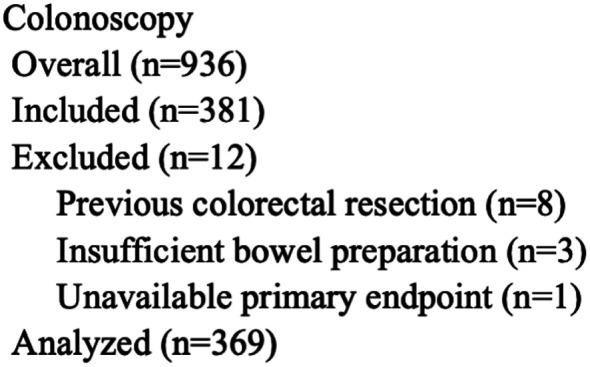
Flowchart of the eligible patients.

Of the 936 patients between May 2021 and April 2022, 381 patients met the inclusion criteria. From these, 12 patients were excluded: 8 due to previous colonic resections, 3 due to insufficient bowel preparations, and 1 due to unavailable primary endpoint. Finally, we analyzed the data of 369 patients (Figure 1). The characteristics of these participants are summarized in Table 1. The mean age and BMI were 68.0 ± 12.4 years and 24.1 ± 5.2 kg/m^2^, respectively. The cohort included 205 men (55.6%), 175 patients (47.4%) with previous CS, 68 (18.4%) with previous abdominal surgery, 59 (16.0%) currently smoking, 128 (34.7%) habitually drinking, and 44 (11.9%) on antiplatelet/coagulant medication. Quantitative and qualitative FIT were performed on 248 (67.2%) and 121 (32.8%) patients, respectively. The number of (−), (−/−), (+), (+/−), and (+/+) FIT results was 7 (1.9%), 24 (6.5%), 50 (13.6%), 204 (55.3%), and 84 (22.8%), respectively. Of these, the patients with at least 1 positive (+) FIT result were 338 (91.6%). The CS examinations were performed as follows: 44 (11.9%) by trainees under supervision, 337 (91.3%) with antispasmodic agents, 69 (18.7%) under sedative agents, and 270 (73.2%) with CS attachment; mean BBPS was 8.8 ± 0.9. Cecum intubation was obtained in 367 patients (99.5%). The mean t-INT and t-OBS times were 13 minutes 48 seconds (±8 minutes 59 seconds) and 14 minutes 3 seconds (±7 minutes 52 seconds), respectively. The mean t-OBS/t-INT ratio was 1.37 ± 1.06. The mean intervention time was 4 minutes 3 seconds (±6 minutes 41 seconds). The total duration was 31 minutes 55 seconds (±16 minutes 29 seconds).

Post-CS nausea caused by pentazocine was recognized in 2 patients (0.5%). Finally, 226 patients (61.2%) had adenoma, and 21 (5.6%) had carcinoma. Details of the total 713 adenomas were as follows: a mean optical size of 5.6 ± 4.3 mm, 672 I-s polyps (94.2%), 33 I-p polyps (4.6%), 3 II-a polyps (0.4%), and 5 II-c polyps (0.7%).

### Stratified Time-Related Indicators and Adenoma Detection Rate

We calculated an appropriate adenoma detection cutoff value for t-OBS/t-INT ratio of 1.0 using the ROC curve. The area under the curve (AUC) was 0.64 (95% CI: 0.58-0.70). We applied the well-known cutoff value for t-OBS of 6 minutes^4, 9^ in this analysis. We stratified by the time-related indicators t-OBS along with t-OBS/t-INT ratio, respectively, into 4 categories as follows: category 1, <6.0 minutes and <1.0; category 2, <6.0 minutes and ≥1.0; category 3, ≥6.0 minutes and <1.0; and category 4, ≥6.0 minutes and ≥1.0.

In the univariate analysis, ADR in categories 3 and 4 were 64.5% (60/93) and 76.9% (130/169), respectively, with significant differences compared to category 1 (*P* < .001 in both comparisons). Furthermore, univariate analyses were performed for other variables of interest, including age, sex, FIT result, previous CS, previous abdominal surgery, smoking status, drinking status, BMI, antiplatelet/anticoagulant, endoscopist skill, sedation, antispasmodic agents, and CS attachment. The BBPS score of 8.8 ± 0.9 in this cohort was excellent, as shown in Table 1. The BBPS scores of categories 1, 2, 3, and 4 were 8.8 ± 1.1, 8.6 ± 1.3, 8.7 ± 1.2, and 8.9 ± 0.4, respectively, without significant between-group differences (*P* = .37). Therefore, we excluded this parameter from the univariate analysis. Age ≥53 years (66.9%, 214/320, *P* < .001), habitual alcohol intake (72.7%, 93/128, *P* = .001), and CS attachment usage (66.7%, 180/270, *P* < .001) were determined as significant factors for ADR (Table 2).

In the logistic regression analysis for these 4 significant factors, age ≥53 years (OR: 4.86, 95% CI: 2.25-10.52), habitual alcohol intake (OR: 2.26, 95% CI: 1.33-3.82), time-related indicators of category 3 (OR: 3.66, 95% CI: 1.81-7.45), and category 4 (OR: 5.60, 95% CI: 2.92-10.73) were revealed as significant positive factors for ADR (Table 2).

### Stratified Time-Related Indicators and Number of Adenomas

**Figure 2.**
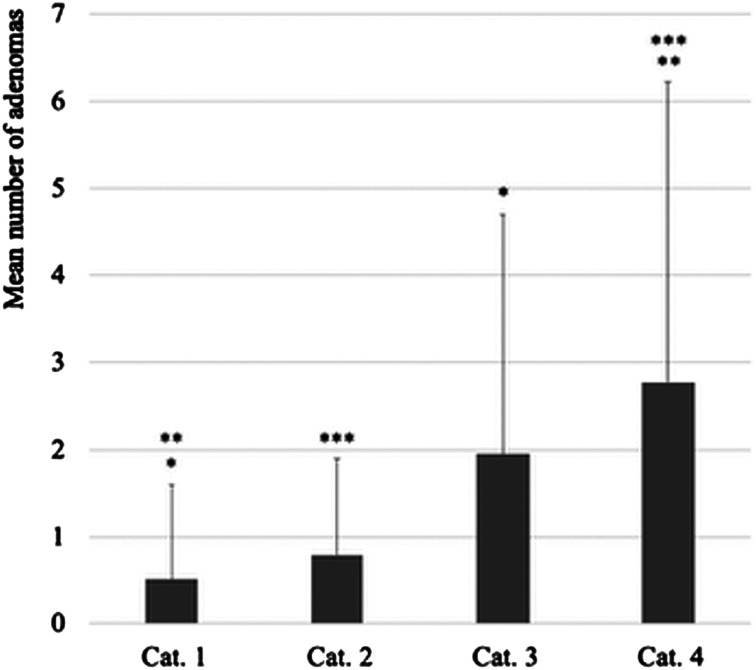
Stratified time-related indicators and number of adenomas. The number of adenomas in each category was as follows: 0.51 ± 1.09, 0.79 ± 1.11, 1.95 ± 2.74, and 2.77 ± 3.46 in categories 1 to 4, respectively, with significant differences between categories 1 and 3, 1 and 4, and 2 and 4 (*P* < .001 in all comparisons). *Category 1 compared with 3; **category 1 compared with 4; ***category 2 compared with 4.

The mean number of adenomas in each stratified time-related indicator category was as follows: 0.51 ± 1.09 in category 1, 0.79 ± 1.11 in category 2, 1.95 ± 2.74 in category 3, and 2.77 ± 3.46 in category 4, with significant differences between categories 1 and 3, categories 1 and 4, and categories 2 and 4 (*P* < .001 in all comparisons) (Figure 2).

### Statistical Power Analysis

This study’s statistical power (1 – *β*) was calculated as 0.999 using parameters obtained from the chi-square test for the primary outcome: sample size = 369, *α* error = 0.05, degrees of freedom = 3, and effect size = 0.382.

## Discussion

This article clarifies the impact of time-related indicators, namely the composite of t-OBS and t-OBS/t-INT ratio, on adenoma detection during the second CS surveillance following the FIT test. The t-OBS ≥6.0 minutes is a well-known, important positive influential factor on ADR.^4, 9^ In our study, when t-OBS was combined with the t-OBS/t-INT ratio, category 3 (t-OBS ≥6.0 minutes, t-OBS/t-INT ratio <1.0) had significantly increased ADR when compared with category 1 (t-OBS <6.0 minutes, t-OBS/t-INT ratio <1.0), with an OR of 3.66. Moreover, the t-OBS/t-INT ratio ≥1.0 additionally improved ADR. Category 4 (t-OBS ≥6.0 minutes, t-OBS/t-INT ratio ≥1.0) had a significantly greater ADR compared to category 1 (OR: 5.60). These results provide an educational message for CS examiners: “inspection over 6 minutes” along with “scope withdrawal time exceeding the intubation one” represent the best time-related indicators of an increased ADR. However, we perform CS within a strict daily time frame of about 20-30 minutes for each CS. When examiners spend a long time for scope intubation, they usually make up for the delay during the observation. This situation harbors the risks of an indiscriminate scope maneuver, an omitted inspection of the folds’ back side, and a skipped water irrigation for microbubbles/remnants. In such situations, it is not useful to achieve t-OBS/t-INT ratio ≥1.0. However, at the very least, the examining endoscopists should aim to maintain observation timings within category 3 (t-OBS ≥6.0 minutes, t-OBS/t-INT ratio <1.0); otherwise, they might risk leaving adenomas undetected.

In our study, the effect of cap-assisted colonoscopy on ADR was equivocal after logistic regression analysis. In previous studies, the effect had also been conflicting.^21- 23^ However, this method potentially increases the ADR of adenomas ≤5 mm (OR: 1.53, 95 % CI: 1.13-1.71).^23^

Our results show that among lifestyle or environmental factors such as age, sex, smoking status, drinking status, and BMI, only age ≥ 53 (OR: 4.86) and current drinking status (OR: 2.26) were significant risk factors for adenomas. Since previous studies regarding risk factors for adenomas were sparse and inconsistent,^24^ a large comprehensive analysis estimating risk factors for adenomas was conducted,^24^ which showed current smoking status as a strong risk factor for serrated adenoma (OR: 2.52) but weak for conventional adenoma (OR: 1.13).^24^ In our study, most lesions were conventional adenomas (serrated adenoma: 1.5%, 11/713, data not shown). This biased adenoma population and weak statistical power resulted in an insignificant effect of smoking status on ADR. The BMI ≥35 kg/m^2^ is a risk factor for both adenomas (OR: 1.34 for serrated adenoma; OR: 1.07 for conventional adenoma) compared to BMI <25 kg/m^2^.^24^ However, 75% of our study population was distributed around a BMI of 25 kg/m^2^, leading to an insignificant result. Alcohol consumption is a risk factor for both adenomas (OR: 1.33 and 1.17).^24^ In our study, alcohol consumption was also a risk factor. Needless to say, advanced age is a risk factor for adenomas.^25^

The FIT result in our study did not significantly increase ADR. This result might come from the relatively low sensitivity (6.0%-56.0%) of FIT for adenoma.^4^ Previous CS was also not significantly correlated with increased ADR. The reasons could be as follows: a clean colon for adenoma was not confirmed after a preceding CS in any of the subjects, and the interval between each CS was not stratified in this study. The examiner’s prior CS experience is also not an important factor to increase ADR, as it has already been advocated that “feedbacking/benchmarking of ADR” is clinically more important than “experience/number of CS” to improve ADR in each examiner.^26^

Category 4 (t-OBS ≥6.0 minutes with t-OBS/t-INT ratio ≥1.0) revealed a significantly increased ADR of 76.9%. The water exchange technique will contribute to further improving ADR; however, the drawback of this technique is the longer cecum t-INT.^16^ Recently, advancing technology has allowed the introduction of artificial intelligence-assisted colonoscopy to increase ADR.^17^ Further study including these methods is warranted to confirm our results. However, the essence of colonoscopy is slow scope movement during the withdrawal phase and diligent inspection beyond every haustrum.

This study had several limitations. First, this is a single-center retrospective study. Second, all patients are Japanese; in other words, there is no racial diversity in the study population. Third, survey subjects are limited to the average CRC-risk population with FIT results. Fourth, this study was performed using various colonoscopes with neither water immersion/exchange techniques nor artificial intelligence detection software.^16, 17^

This study’s data allow us to conclude that in the quality examination of CS, the combination of time-related indicators t-OBS ≥6.0 minutes and t-OBS/t-INT ratio ≥ 1.0 significantly increases ADR. We believe that this benchmark will become one of the CS quality indicators in the second surveillance following the FIT.

## Data Availability Statement:

The datasets generated and analyzed during the current study are available in the UMIN individual case data repository, at https://www.umin.ac.jp/icdr/index-j.html, with the registered number UMIN000049187.

## Figures and Tables

**Table 1.** Baseline Characteristics of the Participants (n = 369)

Age, years, mean (SD)	68.0 (12.4)
Sex, male, n (%)	205 (55.6)
Previous CS, yes, n (%)	175 (47.4)
FIT	
Quantitative, n (%)	248 (67.2)
Qualitative, n (%)	121 (32.8)
FIT result	
Negative, n (%)	31 (8.4)
(-), n (%)	7 (1.9)
(-/-), n (%)	24 (6.5)
Positive, n (%)	338 (91.6)
(+), n (%)	50 (13.6)
(+/-), n (%)	204 (55.3)
(+/+), n (%)	84 (22.8)
Previous abdominal surgery, yes, n (%)	68 (18.4)
Smoking, current, n (%)	59 (16.0)
Drinking, habitual, n (%)	128 (34.7)
Antiplatelet/anticoagulant, yes, n (%)	44 (11.9)
BMI, kg/m^2^, mean (SD)	24.1 (5.2)
Endoscopist skill, trainee, n (%)	44 (11.9)
Antispasmodic agents, yes, n (%)	337 (91.3)
Sedative agents, yes, n (%)	69 (18.7)
Bowel preparation, yes, n (%)	369 (100)
BBPS, score, mean (SD)	8.8 (0.9)
CS attachment, yes, n (%)	270 (73.2)
Cecum intubation, yes, n (%)	367 (99.5)
Duration	
t-INT, min:s, mean (SD)	13:48 (8:59)
t-OBS, min:s, mean (SD)	14:03 (7:52)
Intervention time, min:s, mean (SD)	4:03 (6:41)
Total time, min:s, mean (SD)	31:55 (16:29)
t-OBS/t-INT, mean (SD)	1.37 (1.06)
Adverse events, nausea, n (%)	2 (0.5)
Adenoma	
Total pts, n (%)	226 (61.2)
Total adenomas, n	713
Size, mm, mean (SD)	5.6 (4.3)
I-s, n (%)	672 (94.2)
I-p, n (%)	33 (4.6)
II-a, n (%)	3 (0.4)
II-c, n (%)	5 (0.7)
Carcinoma, total pts, n (%)	21 (5.6)

BBPS, Boston bowel preparation scale; BMI, body mass index; CS, colonoscopy; FIT, fecal immunochemical test; min, minutes; N (n), number; pts, patients; s, seconds; SD, standard deviation; t-INT, intubation time; t-OBS, observation time.

**Table 2.** Predictive Factors for Adenoma Detection Rate

	Adenoma detection		Univariate	Multivariate		
	n/N, %		*P*	*P*	Odds ratio (95% CI)	
Age						
<53 years	12/49	(24.5)	<.001*	<.001*	4.86	(2.25–10.52)
≥53 years	214/320	(66.9)				
Sex						
Female	97/164	(59.1)	.459			
Male	129/205	(62.9)				
FIT result						
Negative	16/31	(51.6)	.250			
Positive	210/338	(62.1)				
Previous CS						
Yes	114/175	(65.1)	.145			
No	112/194	(57.7)				
Previous abdominal surgery						
Yes	40/68	(58.8)	.650			
No	186/301	(61.8)				
Smoking						
Never, ex	187/310	(60.3)	.404			
Current	39/59	(66.1)				
Drinking						
Not-habitual	133/241	(55.2)	.001*	.002*	2.26	(1.33–3.82)
Habitual	93/128	(72.7)				
BMI						
<25.3	148/255	(58.0)	.059			
≥25.3	78/114	(68.4)				
Antiplatelet/anticoagulant						
Yes	29/44	(65.9)	.499			
No	197/325	(60.6)				
Endoscopist skill						
Trainee	30/44	(68.2)	.314			
Not trainee	196/325	(60.3)				
Sedation						
Yes	41/69	(59.4)	.730			
No	185/300	(61.7)				
Antispasmodic agents						
Yes	210/337	(62.3)	.172			
No	16/32	(50.0)				
CS attachment						
Yes	180/270	(66.7)	<.001*	.061	1.68	(0.98–2.88)
No	46/99	(46.5)				
t-OBS, t-OBS/ t-INT ratio						
Cat 1, <6.0 min, <1.0	22/74	(29.7)	Ref.			
Cat 2, <6.0 min, ≥1.0	14/33	(42.4)	.202	.258	1.70	(0.68–4.24)
Cat 3, ≥6.0 min, <1.0	60/93	(64.5)	<.001*	<.001*	3.66	(1.81–7.45)
Cat 4, ≥6.0 min, ≥1.0	130/169	(76.9)	<.001*	<.001*	5.60	(2.92–10.73)

ADR, adenoma detection rate; BMI, body mass index; Cat, category; CI, confidence interval; CS, colonoscopy; FIT, fecal immunochemical test; min, minutes; N (n), number; Ref., reference; t-OBS, observation time; t-INT, intubation time.

*P < .05
